# Draft genome assembly for the purple-hinged rock scallop (*Crassadoma gigantea*)

**DOI:** 10.1186/s12863-025-01330-5

**Published:** 2025-05-28

**Authors:** Hayley Goss, Paige Miller, Susan F. Zaleski, Robert J. Miller, Donna M. Schroeder, Henry M. Page

**Affiliations:** 1 Marine Science Institute, University of California Santa Barbara, Santa Barbara, CA 93106 USA; 2 Bureau of Ocean Energy Management, 760 Paseo Camarillo, Suite 102, Camarillo, CA 93010 USA

**Keywords:** Draft genome, Genome assembly, Purple-hinged rock scallop, Crassadoma gigantean

## Abstract

**Objectives:**

This genomic sequence for the purple-hinged rock scallop, *Crassadoma gigantea*, is a substantial improvement over currently available NCBI genomes for the species and will be an important resource for future genomic research, including ongoing and future population genetic studies. Purple-hinged rock scallops are found along the west coast of North America with a native range from Northern Alaska to Northern Mexico and found to depths of up to ~ 50 m. While not commercially harvested, this species is harvested recreationally and is a candidate of interest for aquaculture and conservation.

**Data description:**

The draft genome for C. gigantea is 817.3 MB, containing 7,183 scaffolds (contig N50 = 287.9 Kb, scaffold N50 = 965.5 Kb). Benchmarking Universal Single-Copy Orthologs (BUSCO) analysis using the 5295 genes in the mollusca_odb10 database had a 93.3% completeness value (92.7% single, 0.6% duplicated). Repeat elements made up 32.23% of the genome. MetaEuk reference-based discovery identified and annotated 23,409 unique protein sequences. Functional annotation was completed by Pannzer2. This assembly will contribute to the ongoing population genetic research on this species.

## Objective

The purple-hinged rock scallop, *Crassadoma gigantea*, is a member of the family Pectinidae and closely related to *Mizuhopecten yessoensis*, *Chlamys islandica*, and *Mimachlamys varia* (the Japanese weathervane, Iceland and variegated scallop, respectively) [1, 2]. *C. gigantea* is widely distributed along the west coast of North America. Like other scallops, *C. gigantea* has a two-phase lifecycle comprising an approximate 30-day pelagic larval stage and a sessile adult stage [3]. *C. gigantea* has a free-living stage following recruitment, during which they can temporarily attach to the substratum with byssal threads; unlike most scallops, this species then has a permanent attachment stage and cements fully to the substratum. *C. gigantea* can be found on subtidal rocky reefs, offshore oil and gas platforms, piers, and other artificial habitats to a depth of approximately 50 m. Because adult *C. gigantea* are sessile and have a 30-day pelagic larval duration (PLD) typical of many marine species, this scallop is an ideal model organism for studying population connectivity of reef invertebrates via planktonic dispersal among rocky reefs and artificial habitats.

*C. gigantea* has a patchy distribution and is highly susceptible to overharvesting and local depletion. These characteristics result in the species being currently unsuitable for commercial harvest [4]. However, the large abductor muscle, strong market potential, and broad geographic distribution make it a candidate for ongoing and future aquaculture endeavors [4–6]. Generating an improved genome assembly for this species will be useful for both aquaculture [7] and ongoing population genetic studies along the West Coast. Incorporating the available draft genomes into population genetic work helps correct for low-level sequencing errors in reads, improve population genetic inferences, and better enable SNP detection compared to traditional de novo techniques [8–10]. Its inclusion in future studies will enhance our understanding of this species’ population structure, connectivity, and adaptation to environmental variability.

## Data description

We collected one *Crassadoma gigantea* individual from each of three locations (Diablo Reef, Naples Reef, offshore oil and gas Platform A in the Santa Barbara Channel (Table 1, Data file 1, 2). High molecular weight DNA was obtained from mantle tissue of each individual using a DNeasy^®^ Blood and Tissue Kit protocol with minor adjustments to incubation times and buffer quantities (Qiagen, USA). Short-read libraries were prepared on a DNBSEQ G400 (945.85 million reads, read length 150 bp) using DNA extracted from samples collected at Diablo and Naples Reef (BGI Genomics). The Platform A sample was used to generate a long-read library by SNPsaurus, LLC (Eugene, OR) using the PacBio sequencing platform (2,919,452 reads, mean sequence length of ~ 15,000 bp). For additional details regarding methods, see Data Table 1, File 11.

**Table 1:** Overview of data files/data sets

Label	Name of data file/data set	File types(file extension)	Data repository and identifier (DOI or accession number)
*Data file 1*	Table 1, *Data File 1. Image of in situ Crassadoma gigantea*	*Microsoft Word (.docx)*	Figshare [11]: 10.6084/m9.figshare.27237210.v3
*Data file 2*	Table 1, *Data File 2. Map of Southern California sampling area*	*Microsoft Word (.docx)*	Figshare [11]: 10.6084/m9.figshare.27237210.v3
*Data file 3*	Table 1, *Data File 3. Confidence Scores for RagTag Scaffolding*	*Microsoft Word (.docx)*	Figshare [11]: 10.6084/m9.figshare.27237210.v3
*Data file 4*	Table 1, *Data File 4. Assembly statistics for the six genome assemblies*	*Microsoft Word (.docx)*	Figshare [11]: 10.6084/m9.figshare.27237210.v3
*Data file 5*	Table 1, *Data File 5. Cumulative coverage plot comparing different assemblies*	*Microsoft Word (.docx)*	Figshare [11]: 10.6084/m9.figshare.27237210.v3
*Data file 6*	Table 1, *Data File 6. BUSCO comparisons of all assemblies*	*Microsoft Word (.docx)*	Figshare [11]: 10.6084/m9.figshare.27237210.v3
*Data set 7*	Table 1, *Data File 7. DataSnail plot summary of assembly statistics for Crassadoma gigantea*	*Microsoft Word (.docx)*	Figshare [11]: 10.6084/m9.figshare.27237210.v3
*Data file 8*	Table 1, *Data File 8. Percentage and summary of interspersed repeat elements*	*Microsoft Word (.docx)*	Figshare [11]: 10.6084/m9.figshare.27237210.v3
*Data file 9*	Table 1, *Data File 9. Pannzer2 results. Gene ontology output table*	*Excel file (.xlsx)*	Figshare [11]: 10.6084/m9.figshare.27237210.v3
*Data file 10*	Table 1, *Data File 10. Pannzer2 results*,* Gene ontology description and counts*	*Excel file (.xlsx)*	Figshare [11]: 10.6084/m9.figshare.27237210.v3
*Data file 11*	Table 1, *Data File 11. Complete methodology for C. gigantea assembly*	*Microsoft Word (.docx)*	Figshare [11]: 10.6084/m9.figshare.27237210.v3
*Data set 1*	*Genomic Assembly of C. gigantea*	*Fasta file (.fa)*	NCBI GenBank Database: JBLBVN000000000 http://identifiers.org/nucleotide:JBLBVN000000000 [12]

We created three de novo assemblies. A hybrid assembly was performed using MASURCA [13, 14], and two long read assemblies were produced, one with WTDBG2 [15] and one with CANU [16]; long read assemblies were polished using NextPolish [17]. Due to the highly fragmented nature of the assemblies, we ran an additional RagTag reference-guided [18, 19] step on each of the assemblies, using published genomes from two closely related species, *Mimachlamys varia *[20], and *Mizuhopecten yessoensis *[21]. We assessed if these assemblies were appropriate for reference-based scaffolding using the confidence scores and localization stats output from RagTag and incorporated the RagTag merge feature to reduce the likelihood of false joins and help correct potential misassemblies (Table 1, Data File 3) [18, 19]. Contaminants were identified and removed using the NCBI Foreign Contamination Screen and Benchmarking Universal Single-Copy Orthologs (BUSCO) [22] and the BBtools [23] stats.sh script was used to evaluate each assemblies’ completeness and quality. The RagTag assembly software tools improved all de novo assemblies. Individual statistics for each assembly can be found in Table 1, Data Files 4, 5, 6. The WTDBG2/NextPolish/Ragtag pipeline performed best and resulted in an 817,168,913 bp genome comprised of 7,183 scaffolds and 8,611 contigs (contig N50 = 287 Kb, scaffold N50 = 965 Kb). BUSCO completeness was 93.3% (92.7% single, 0.6% duplicated), with 1.4% fragmented and 5.3% missing (Table 1, Data File 7). This is a marked improvement over the currently available NCBI *C. gigantea* genome, which is 902,550,592 bp, with an N50 scaffolding length of 2.9 kb, and a BUSCO completeness of 25.9%; 25.3% single, 0.6% duplicated (Table 1, Data File 4, 5, 6) [24]. The WTDBG2 assembly was used for all downstream analyses.

Interspersed repeat elements were identified by RepeatModeler2 [25] and RepeatMasker [26] using the rebase Metazoa database. 263,454,073 bp (32.23%) of the genome was identified as repeats, with the most common being Unclassified (14.08%) and Retroelements (11.68%) (Table 1, Data File 8). Metaeuk [27], used for gene discovery and annotation, identified 23,409 individual protein sequences. PANNZER2 [28] was used to functionally annotate all identified protein sequences; the top two gene ontologies for biological processes, cellular components, and molecular function were process/regulation, membrane/complex, and binding/ion, respectively (Table 1, Data Files 9,10).

## Limitations

Reference scaffolding using divergent species can introduce a risk of misassemblies. While the confidence score distribution plots support using these two species for reference scaffolding, if needed the available genome may also be broken down back to its original 8,611 contigs using several off-the-shelf scaffold breaking pipelines. (817.161 MB, 8611 contigs, N50 = 287.862 KB).

The assembled draft genome size for this species is smaller than the expected size based on feulgen imagery analysis and Kmer analysis. This may be due to an over-collapsing of repetitive regions. A similar phenomenon was observed during the yesso scallop assembly [21]. This *C. gigantea* assembly size is similar to four previously reported scallop genome sizes in the Pectinidae family, which range between 724 and 988 Mb [29].

## Data Availability

The genome described in this Data Note can be freely and openly accessed on NCBI GenBank under the accession number JBLBVN000000000. Please see Table 1 and references [11] for details and links to the data. The genome assembly is available at https://www.ncbi.nlm.nih.gov/assembly/JBLBVN000000000.

## Abbreviations

Kb: Kilobases
Gb: Gigabases
MP: Million basepairs
bp: Basepair
BUSCO: Benchmarking Universal Single-Copy Orthologs
PLD: Pelagic Larval Duration
