# Publisher Correction: Draft genome assembly for the purple-hinged rock scallop (Crassadoma gigantea)

**DOI:** 10.1186/s12863-025-01339-w

**Published:** 2025-07-22

**Authors:** Hayley Goss, Paige Miller, Susan F. Zaleski, Robert J. Miller, Donna M. Schroeder, Henry M. Page

**Affiliations:** 1 Marine Science Institute, University of California Santa Barbara, Santa Barbara, CA 93106 USA; 2 Bureau of Ocean Energy Management, 760 Paseo Camarillo, Suite 102, Camarillo, CA 93010 USA

**Publisher Correction** **:** **BMC Genomic Data 26, 39 (2025)**

**https://doi.org/10.1186/s12863-025-01330-5**

Following publication of the original article, it came to the journal's attention that the row of Table 1 beginning data file 3 was incomplete: 'Confidence Scores for RagTag Scaffolding' and 'Microsoft Word (.docx)' were missing. The table has since been corrected; please refer to the current version of the table. The publisher thanks you for reading this erratum and apologizes for any inconvenience caused.

